# Abrogated RANKL expression in properdin-deficient mice is associated with better outcome from collagen-antibody-induced arthritis

**DOI:** 10.1186/ar3926

**Published:** 2012-07-25

**Authors:** Petya Dimitrova, Nina Ivanovska, Lyudmila Belenska, Viktoriya Milanova, Wilhelm Schwaeble, Cordula Stover

**Affiliations:** 1 Department of Immunology, Institute of Microbiology, 26 Georgi Bonchev St., Sofia, 1113, Bulgaria; 2 Department of Infection, Immunity and Inflammation, University of Leicester, Maurice Shock Medical Sciences Building, University Road, Leicester, LE1 9HN, UK

## Abstract

**Introduction:**

Properdin amplifies the alternative pathway of complement activation. In the present study, we evaluated its role in the development of collagen antibody-induced arthritis (CAIA).

**Methods:**

Arthritis was induced by intraperitoneal injection of a collagen antibody cocktail into properdin-deficient (KO) and wild-type (WT) C57BL/6 mice. Symptoms of disease were evaluated daily. The degree of joint damage was assessed histologically and with immunostaining for bone-resorption markers. Phenotypes of cell populations, their receptor expression, and intracellular cytokine production were determined with flow cytometry. Osteoclast differentiation of bone marrow (BM) precursors was evaluated by staining for tartrate-resistant acid phosphatase (TRAP).

**Results:**

Properdin-deficient mice developed less severe CAIA than did WT mice. They showed significantly improved clinical scores and downregulated expression of bone-resorption markers in the joints at day 10 of disease. The frequencies of Ly6G^+^CD11b^+ ^cells were fewer in BM, blood, and synovial fluid (SF) of KO than of WT CAIA mice. The receptor activator of nuclear factor κB ligand (RANKL) was downregulated on arthritic KO neutrophils from BM and the periphery. Decreased C5a amounts in KO SF contributed to lower frequencies of CD5aR^+^-bearing neutrophils. In blood, surface C5aR was detected on KO Ly6G^+ ^cells as a result of low receptor engagement. Circulating CD4^+ ^T cells had an altered ability to produce interleukin (IL)-17 and interferon (IFN)-γ and to express RANKL. In KO CAIA mice, decreased frequencies of CD4^+ ^T cells in the spleen were related to low CD86 expression on Ly6G^high^CD11b^+ ^cells. Arthritic KO T cells spontaneously secreted IFN-γ but not IL-17 and IL-6, and responded to restimulation with less-vigorous cytokine production in comparison to WT cells. Fewer TRAP-positive mature osteoclasts were found in KO BM cell cultures.

**Conclusions:**

Our data show that the active involvement of properdin in arthritis is related to an increased proinflammatory cytokine production and RANKL expression on immune cells and to a stimulation of the RANKL-dependent osteoclast differentiation.

## Introduction

Rheumatoid arthritis (RA) is an autoimmune disorder leading to chronic inflammation of the joints and subsequent erosion of cartilage and bone. Because RA is a complex heterogeneous disease, different animal models are exploited to understand better its mechanisms of development and to find effective approaches for treatment. In one such model, arthritis is induced by the injection of antibodies that bind to specific triple helical epitopes of collagen II [1]. The additional administration of lipopolysaccharide (LPS) 3 to 5 days after antibody introduction synchronizes the course and increases the severity of the disease [2]. Several studies have optimized the doses of antibody cocktail (from 2 to 9 mg per mouse) and of LPS (25 to 50 μg/mouse) [2-4]. Arthritis develops rapidly within 24 to 72 hours and is characterized by massive cellular infiltration, synovitis, and cartilage/bone erosion. The disease is induced in collagen-induced arthritis (CIA)-susceptible DBA/1 and B10.RIII mice with a high incidence and in CIA-resistant BALB/c and C57BL/6 mice [3].

Complement cascade is initiated by three major pathways: classic, alternative, and lectin pathways. Alternative complement pathway (AP) starts with a spontaneous hydrolysis of native C3 to C3(H_2_O) followed by binding to factor B and formation of AP C3 convertase [C3(H_2_O)3b], which generates C3b. C3b quickly attaches to nearby surfaces and further binds properdin or factor H, forming AP C5 convertase (C3b)_2_Bb. AP can be triggered by interaction of properdin with C3b(2)-IgG complexes present in serum [5] or by the attachment of properdin to cell surfaces [6]. Properdin increases the stability of C3 convertase and enhances the assembly of the enzyme on a target surface, resulting in an increased half-life of the enzyme and the amplification of AP [7]. The factor is expressed in peripheral blood T cells [8] and monocytes [9] and is released from neutrophil granules after stimulation [10]. The latter process is controlled by a particular C3 fragment in a way that intravascular AP activation is limited, but AP is augmented at sites of inflammation [11]. The central role of AP for arthritis development has been shown in CAIA and CIA [12,13]. The amelioration of disease activity has been observed in factor B- or C3-deficient mice with CAIA but not in mice lacking C4, C1q, mannose-binding lectin (MBL) A, C, or both C1q and MBL [14]. The injection of collagen antibodies into C5-deficient mice does not promote disease pathology [15].

Neutrophils are key players in the pathogenesis of CAIA because their depletion with anti-Gr1 antibody abolishes the severity of the disease to a larger extent [3]. Neutrophil involvement in the pathologic process is mediated by the recognition of the immune complexes by Fcγ receptors (FcγRs). Murine neutrophils express three activating receptors: the high-affinity receptor FcγRI (CD64), the low-affinity FcγRIII (CD16), and the intermediate-affinity FcγRIV, and one inhibitory low-affinity receptor, FcγRIIb (CD32) [16]. The engagement of activating FcγRs induces calcium mobilization and cytokine production and promotes phagocytosis, degranulation, and reactive oxygen species generation [17]. Mice with FcRγ-chain deficiency are completely protected from CAIA development; the disease progression is partially suppressed in FcγRIII-deficient mice [18]; and arthritis was more severe in FcγRIIa-transgenic mice [19]. The FcγRIIb delivers inhibitory signals after co-ligation with activating receptors and regulates the extent of cell activation [20]. FcγRIIb^-/- ^mice develop severe joint inflammation and bone destruction [21,22]. It is considered that the balance between activating and inhibitory FcγRs is a mechanism for terminating activation responses in arthritis [23].

Recently, it was shown that RA neutrophils upregulate RANKL in response to different stimuli, like LPS [24]. RANKL is an important factor regulating activation and differentiation of osteoclasts and, in turn, bone resorption. Beside neutrophils, B lymphocytes from RA patients express mRNA for RANKL [25]. RANKL-positive T cells are detected in gouty arthritis [26]. The expression of RANKL by different cell populations provides the source of soluble RANKL in synovial fluid and serum in favor of osteoclastogenesis and bone resorption. Currently, RANK/RANKL interaction is a target for immunotherapy of bone diseases [27].

Previously, we investigated the role of properdin for the development of zymosan-induced arthritis (ZIA) [28]. At the initial phase of disease, cell infiltration in the joints, cartilage proteoglycan loss, synovial TNF-α and soluble RANKL levels are similar in wild-type and properdin-deficient mice. The lack of properdin, however, attenuates the local generation of C5a and IL-6 in synovial fluid and alters IFN-γ production, IFN-γ receptor expression, and signal transducer and activator of transcription (STAT)1 signaling in ZIA splenocytes. Increased proteoglycan loss at a late phase of disease (day 30) in properdin-deficient mice was observed, along with decreased serum levels of circulating zymosan-specific IgG antibodies, reduced STAT1 joint expression, and enhanced C5a receptor (C5aR) staining in cartilage. However, in this experimental model, arthritis develops via zymosan-mediated activation of classic and alternative complement pathways and the engagement of Toll-like receptor 2 (TLR2) on monocytes and neutrophils. To study further the significance of properdin for the initiation and progression of arthritis, we used the CAIA model, in which immune complex formation and the activation of immune cells through FcγRs contribute to the disease pathology. In the present study, we evaluated receptor expression and cytokine production in immune cells (neutrophils, monocytes, T cells), histopathologic changes, and the expression of bone-resorption markers in the joints of properdin-deficient and wild-type mice with CAIA. We showed that RANKL appears to be an important factor in the mechanism of properdin action.

## Materials and methods

### Mice

Properdin-deficient and wild-type mice, 20 to 25 g body weight, 10- to 12-week-old, male and female, were used in our experiments. Properdin-deficient mice were genetically engineered through gene-specific targeting to be deficient of properdin and were shown to lack properdin in their serum [29]. The novel line is designated Cfp^tm1Cmst^, and homo- and hemizygous mice are termed KO. Wild-type C57BL/6 mice were from the same fully backcrossed line. Animals were maintained in a specific pathogen-free environment and had free access to water and standard chow. All experiments were conducted in accordance with the International and National Guidelines for the Care and Use of Laboratory Animals and were approved by the Animal Care Committee at the Institute of Microbiology, Sofia.

### Experimental design

A cocktail of four monoclonal antibodies to type II collagen (ArthritoMab; MD Biosciences, Saint Paul, MN, USA; 5 mg/100 μl) was injected intraperitoneally at day 0. The ArtritoMab binds to the epitopes C1^I^, J1, U1, and D3 in the full-length CII fragments (CB8, CB10, and CB11). The internal control group of mice received equal volume of sterile phosphate-buffered saline PBS (pH 7.4) (PBS group). At day 3, all animals were intraperitoneally injected with LPS (*Escherichia coli *055:B5; MD Biosciences; 50 μg/200 μl endotoxin-free water). Mice were examined for the development of arthritis for 10 days. Clinical score was done blindly by using a system based on the number of inflamed joints in front and hind paws, inflammation being defined by swelling and redness at the scale from 0 (no redness and swelling) to 3 (severe swelling with joint rigidity or deformity; maximal score for four paws, 12).

### Collection of synovial fluid and plasma

Synovial fluid was harvested by lavage of the knee cavity with 25 μl of PBS containing 1 m*M *ethylenediaminetetraacetic acid (EDTA; Sigma-Aldrich, Diesenhofen, Germany). After centrifugation, the cell pellets from all samples per group were pooled, counted, and used for flow-cytometry analyses while supernatants were stored at -70°C and assayed for cytokine and C5a content. Blood was collected in heparin tubes (10 U heparin/ml). Plasma was obtained after centrifugation at 350 *g *for 15 minutes at 4°C, stored at -70°C, and used for cytokine and C5a measurements.

### Isolation of bone marrow cells

Tibias and femurs of WT and KO mice were collected in sterile tubes with plastic adapter and then centrifuged at 350 *g *for 30 seconds. The cell pellet was washed twice with PBS containing 1 m*M *EDTA, resuspended at concentrations of 1 × 10^6 ^cells/ml, and used for flow-cytometry analyses.

### Isolation of blood neutrophils and mononuclear cells

Blood was mixed in an equal volume with 6% Dextran T-500 sodium salt (Sigma-Aldrich) diluted in 0.9% NaCl (pH 7.0) and incubated for 40 minutes at room temperature. The upper layer was subjected to gradient centrifugation for 30 minutes at 350 *g*, 22°C. The layer enriched with mononuclear cells was carefully collected, washed with PBS, resuspended at concentrations of 1 × 10^6 ^cells/ml, and used for flow-cytometry analyses or for further purification of CD4^+ ^T cells. The erythrocytes in the pellet were lysed with buffer (155 m*M *NH_4_Cl, 10 m*M *KHCO_3_, 2 m*M *EDTA; pH 7.4) for 3 minutes, neutrophils were washed with PBS, counted, resuspended at concentrations of 1 × 10^6 ^cells/ml, and used for flow-cytometry analyses. Exclusion dye staining with 0.05% Trypan blue showed more than 95% viable cells in isolated populations.

### Isolation of spleen cells

Cell suspensions from KO and WT mice were prepared from freshly removed spleens and after erythrocyte lysis [28]. The population was passed through a sterile strainer with 70-μm nylon mesh pores (BD Falcon; BD Biosciences) and washed with PBS. Splenocytes were resuspended at concentrations of 1 × 10^6 ^cells/ml and then used for flow-cytometry analyses and cytokine assays.

### Isolation of CD4^+ ^T cells

CD4^+ ^T cells were purified by indirect panning of spleen and blood cell populations. Petri dishes were coated with Fc specific antibody against rat IgG (10 μg/ml) for 24 hours at 4°C. Cells (1 × 10^6 ^cells/ml) in 2% fetal calf serum (FCS)/PBS were incubated with rat antibodies against CD14 (rmC5-3), CD16 (clone 2.4G2), CD19 (clone 1D3), and CD8 (clone OX8) (all from BD Pharmingen, BD Biosciences; 0.2 mg/1 × 10^6 ^cells) for 15 minutes at 4°C. After washing with PBS, cell suspension was resuspended in 5 ml 5% FCS/PBS, added to the coated Petri dishes, and incubated for 10 minutes at room temperature. Unbound cells were eluted carefully, centrifuged, and resuspended in sterile complete RPMI-1640 medium (Biowhittaker; Lonza, Basel, Switzerland) containing 5% FCS, 100 U/ml penicillin, 100 μg/ml streptomycin, 2 m*M *L-glutamine, and 25 μ*M *β-mercaptoethanol, and counted. The purity of the cell population was 85% to 90%. CD4^+ ^T cells (1 × 10^6 ^cells/ml) were stimulated in 48-well plates with concanavalin A (ConA; 2 μg/ml; Sigma-Aldrich) and in the presence of murine recombinant IL-2 (10 ng/ml; PeproTech EC, London, UK), or where indicated, with plate-bound anti-CD3 (10 μg/ml; clone 145-2C11; BD Biosciences) and soluble anti-CD28 (2 μg/ml; clone 37.51; BD Biosciences) antibodies. After 48 hours at 5% CO_2_, 37°C, plates were centrifuged at 350 *g *for 10 minutes at 4°C; cells were collected, washed twice, and subjected to flow-cytometry analyses for intracellular production of IL-17 and IFN-γ. Supernatants from splenic CD4^+ ^T cultures were used in enzyme-linked immunosorbent assay (ELISA) for determination of IL-6, IFN-γ, and IL-17 secretion.

### Assay for detection of cytokines and C5a

The levels of IL-17 in synovial fluid, plasma, and culture supernatants and of IL-6 and IFN-γ in the spleen-culture supernatants were determined with ELISA by using commercial kits of PeproTech EC (London, UK) with detection limit of 8 pg/ml and of 20 pg/ml, and of Abcam (Cambridge, UK; detection limit of 16 pg/ml), respectively. The amount of C5a in synovial fluid was evaluated as previously described [30]. The samples were assayed in triplicate. The concentrations of the cytokines and C5a were calculated from a standard curve of the respective recombinant mouse protein, by using Gen5 Data Analysis Software (BioTek Instruments, Bad Friedrichshall, Germany).

### Flow cytometry

Freshly isolated cell populations (1 × 10^5^/sample) were washed with 2% FCS/PBS and incubated with antibodies against CD3 (clone 145-2C11; FITC labeled; BD Pharmingen), CD4 (clone L3/T4; PE-labeled; BD Pharmingen), Ly6G (clone 1A8, FITC or APC-labeled; BioLegend, Uithoorn, Netherlands), CD11b (clone M1-70; Alexa-Fluor 647-labeled; BioLegend), CD69 (clone H1.2F3; APC-labeled; BD Pharmingen), CD14 (clone rmC5-3; PerCP-Cy 5.5-labeled; BD Biosciences), RANKL (clone IK22/5; PE-labeled; BioLegend), C5aR (clone 20/70; PE labeled; BD Biosciences or APC-labeled; BioLegend); C5L2 (human reactivity; clone 1D-M12; PE labeled; BioLegend); FcγRIII/II (clone 2.4G2; FITC-labeled; BD Pharmingen), and IgG isotype controls for 15 minutes at 4°C. After washing with 2% FCS/PBS, the samples were analyzed with flow cytometer (BD LSR II) by using BD FACSDiva v6.1.2 Software (Becton Dickinson GmbH, San Jose, CA, USA). In the experiments determining the expression of C5L2 on mouse peripheral neutrophils, purified anti-C5L2/GPR77 (clone 468705; R&D Systems, Wiesbaden-Nordenstadt, Germany) was incubated for 15 minutes at 4°C followed by washing steps and staining with anti-rat IgG-PE (minimal x reactivity; BioLegend). The isotype monoclonal rat IgG2b (R&D Systems) was used to control staining specificity.

### Intracellular flow cytometry

CD4^+ ^T cells were restimulated with phorbol-12-myristate-13-acetate (PMA; 10 ng/ml; Sigma-Aldrich) and ionomycin (2 μ*M*; Sigma-Aldrich) for 4 hours in the presence of protein-transport inhibitor, monensim (2 μ*M*; Beckton Dickinson). The cells were centrifuged at 350 *g *for 10 minutes, washed twice with PBS, counted, and resuspended at 2 × 10^5 ^cells/ml in PBS. After fixation and permeabilization (BD Cytofix^M ^buffer; BD Perm/Wash^M ^buffer; Becton Dickinson), the cells were washed and stained with BD Pharmingen antibodies against murine IL-17 (clone TC11-18H10; PE-labeled; 0.125 μg/1 × 10^6 ^cells); IL-4 (clone 11B11; APC-labeled; 0.25 5 μg/1 × 10^6 ^cells), and IFN-γ (clone XMG 1.2; FITC labeled; 0.5 μg/1 × 10^6 ^cells), or of isotype controls (APC- or FITC-labeled R3-34 or unlabeled TC11-18H10 antibodies). The samples were incubated for 30 minutes at room temperature in the dark, washed, and used for flow-cytometry analysis.

### Osteoclast differentiation

Bone marrow cells were resuspended at 2 × 10^6^/ml in 10% FCS/MEM medium (Lonza). BM cells were incubated for 1 day with medium containing macrophage colony-stimulating factor (M-CSF; 30 ng/ml; PeproTech). Osteoclast precursors were generated in cultures with M-CSF (30 ng/ml) and RANKL (50 ng/ml; PeproTech EC). After 3 days, fresh medium supplemented with growth factors (M-CSF and RANKL) was added to the cultures. In some experiments, blocking anti-RANKL antibody (5 μg/ml; PeproTech EC) was added at this time. The cells were allowed to differentiate for 3 days and the specific tartrate-resistant acid phosphatase (TRAP) staining was performed, as described [31]. The number of TRAP-positive cells was determined by light microscopy (Leica Microsystems, Wetzlar, Germany) by two independent observers and additionally, by software analyses (ImageJ 1.42; Research Services Branch, NIH, Bethesda, MD, USA) after photo capturing by a DS-Ri1 Nikon camera (Nikon Instruments Europe, Amstelveen, The Netherlands).

### Histologic analyses

Paws were fixed in 10% paraformaldehyde/PBS (pH 7.4), decalcified for 4 days in 5% nitric acid (Sigma-Aldrich), dehydrated in ethanol series and xylene substitute (Tissue-Clear, Sakura Finetek, Tokyo, Japan) and embedded in paraffin. The sections with thickness 6 μm were cut by rotary microtome (Accu-Cut SRM Sacura Finetek). Safranin O and hematoxylin and eosin (H&E) staining was performed. The sections were examined with a light microscope by using 1 × 100 or 1 × 400 lens. Images were captured with a coupled device camera and exported to Adobe Photoshop 7.0 (Adobe Systems, Munich, Germany).

All histologic assessments were performed in a blinded protocol. The degree of injury was graded for infiltration (score 0, normal; score 1, mild infiltration; score 2, moderate infiltration; score 3, marked infiltration; score 4, severe infiltration). Proteoglycan loss was graded as follows: score 0, normal; score 1, minimal loss; score 2, moderate loss; score 3, marked loss; score 4, severe, diffuse loss). Bone erosion was graded as score 0, no abnormality; score 1, small areas of resorption; score 2, more numerous areas of resorption, not readily apparent on low magnification; 3, obvious resorption in trabecular and cortical bone, and lesions apparent on low magnification; score 4, thickness defects in the cortical bone and trabecular bone loss.

### Immunohistochemistry

Immunohistochemistry was used to evaluate the expression of STAT1, STAT3, transforming growth factor (TGF)-β3, RANKL, and C5aR in the joints. After blocking of endogenous peroxidase and unspecific binding, the sections were incubated for 1 hour with antibodies against C5aR (1:50 diluted; BD Biosciences), STAT3 (1:100 diluted, Santa-Cruz Biotechnology, Heidelberg, Germany), STAT1 (1:500 diluted, Santa-Cruz Biotechnology), TGF-β3 (1:50 diluted; Abcam, Cambridge, UK), and RANKL (1:50 diluted; PeproTech EC). Isotype antibodies (with rat or rabbit origin, Abcam) were used as specific controls in the experiments. The sections were washed, and HRP/DAB detection kit (Abcam) was used to detect specific staining.

### Statistical analyses

Statistical analysis was accomplished by using InStat3.0 and GraphicPad Prism 5.0 (GraphPad Software, La Jolla, CA, USA). Data were expressed as mean ± standard deviation (SD). The histologic score and the immunohistochemistry data were analyzed with the Mann-Whitney *U *test. For other data, the differences in the mean values between groups were analyzed with the two-tailed Student *t *test. Differences were considered significant when *P *< 0.05.

## Results

### Reduced manifestation of CAIA in properdin-deficient mice

**Figure 1:**
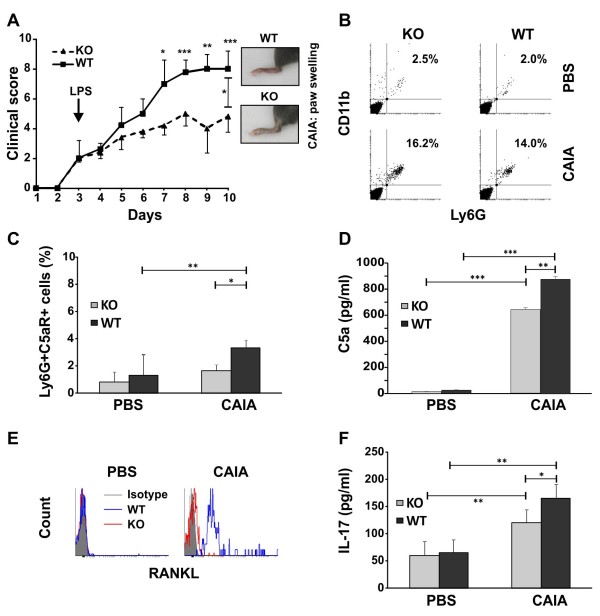
**Clinical evaluation of arthritis and flow-cytometry analyses of properdin-deficient and wild-type synovial neutrophils**. **(A) **Properdin-deficient (KO, *n *= 15) and wild-type (WT, *n *= 15) mice were intraperitoneally injected with a collagen antibodies cocktail followed by administration of LPS at Day 3 (arrow). Photos show the hind-paw swelling of WT and KO mice at Day 10 of arthritis. Clinical score was calculated for each time point by using the semiquantitative grading system. Data are expressed as the mean ± SD. Significant differences of *P < 0.05; **P < 0.01; and ***P < 0.001 between KO and WT groups are indicated, Mann-Whitney *U *test. Line segment with *P < 0.05 shows the statistical significance between WT and KO line curves; Mann-Whitney *U *test. **(B) **The frequencies of Ly6G^+^CD11b^+ ^neutrophils were less in synovial fluid of properdin-deficient than in wild-type mice at Day 10 of arthritis. The data are representative of three separate experiments and show the analyses of the synovial cell pool from five mice/group. **(C) **Lower numbers of Ly6G^+^C5aR^+^-bearing cells and **(D) **decreased amounts of C5a were found in synovial fluid of properdin-deficient mice with arthritis in comparison with wild-type CAIA mice. Data are expressed as the mean ± SD of two experiments involving five mice/group. *P < 0.05; **P < 0.01; and ***P < 0.001; Student *t *test. **(E) **RANKL was expressed on wild-type synovial CAIA neutrophils but not on properdin-deficient arthritic cells. The data are representative of three separate experiments and show the analyses of the synovial cell pool from five mice/group. **(F) **IL-17 production in synovial fluid of wild-type mice was significantly higher than that of properdin-deficient mice at Day 10 of disease. Data represent the mean ± SD of two experiments involving five mice/group. *P < 0.05; **P < 0.01; Student *t *test.

Properdin-deficient mice developed less severe arthritis. We observed significantly attenuated clinical symptoms of the disease in KO mice compared with WT mice after day 7 (Figure 1A). At the end of the experiment (day 10), the clinical score of KO mice decreased by 52.5% in comparison with WT mice (clinical score, KO CAIA group, 4.2 ± 0.8; WT CAIA group, 8.0 ± 1.2; *P *< 0.001, Mann-Whitney *U *test). Histologic examination showed a massive presence of inflammatory cells in the synovial tissue and cartilage of WT mice, which was markedly reduced in KO mice (score for cell infiltration: KO CAIA group, 2.20 ± 0.40; WT CAIA group, 3.00 ± 0.35; *n *= 5; *P *< 0.05). Safranin O staining of joint sections showed weak proteoglycan cartilage loss in WT and KO mice with CAIA (score for proteoglycan loss: KO CAIA group, 0.40 ± 0.20; WT CAIA group, 0.60 ± 0.25).

### Decreased RANKL expression on synovial properdin-deficient arthritic neutrophils

At day 10 of CAIA, we detected higher numbers of SF cells in WT than in KO mice (WT CAIA group, 0.70 × 10^5 ^cells/ml ± 0.02; KO CAIA group, 0.10 × 10^5 ^± 0.01 cells/ml; *n *= 5; *P *< 0.05). CAIA mice showed increased numbers of Ly6G^+^CD11b^+ ^(Figure 1B) and of Ly6G^+^C5aR^+ ^(Figure 1C) neutrophils compared with PBS-injected control mice. The lack of properdin significantly decreased the frequencies of Ly6G^+^C5aR^+ ^cells (Figure 1C) and C5a levels in SF (Figure 1D). The expression of RANKL was higher on WT CAIA neutrophils in comparison with the KO group (Figure 1E). These data suggest that WT synovial Ly6G^+ ^cells can participate more actively in RANKL-dependent processes of osteoclast activation and differentiation. IL-17 enhances inflammation and osteoclastogenesis. Higher amounts of synovial IL-17 were observed in arthritic groups compared with PBS-injected ones (Figure 1F). IL-17 levels in KO CAIA SF were significantly lower than those in WT CAIA mice (Figure 1F). CAIA developed with slightly (nonsignificantly) elevated amounts of plasma IL-17 in WT compared with KO mice (KO CAIA group, 358.8 pg/ml ± 10.2; WT CAIA group, 401.5 pg/ml ± 22.6; *n *= 5; *P *= 0.068).

### Altered RANKL and C5aR expression on properdin-deficient bone marrow and blood arthritic neutrophils

**Figure 2:**
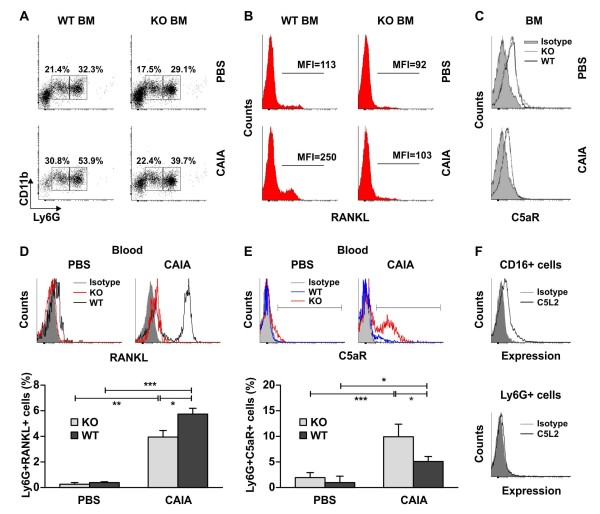
**Flow-cytometry analyses of properdin-deficient and wild-type bone marrow and blood neutrophils**. **(A) **Frequencies of Ly6G^low^CD11b^+ ^and Ly6G^high^CD11b^+ ^cells were less in the BM of properdin-deficient (KO) mice with arthritis than in those of the wild-type (WT) group. The data are representative of three separate experiments involving four mice/group. **(B) **RANKL was expressed on Ly6G^high^CD11b^+ ^cells isolated from wild-type arthritic mice but not on those from properdin-deficient mice with CAIA. The values of the mean fluorescence intensity (MFI) are presented in each histogram. **(C) **Ly6G^high^CD11b^+ ^BM cells from properdin-deficient and wild-type mice expressed C5aR as presented in one individual experiment. **(D) **Histograms show upregulated RANKL expression on arthritic wild-type neutrophils isolated from blood at Day 10 of CAIA. The data on the graph represent the mean ± SD of the percentage of Ly6G^+ ^RANKL^+ ^cells in three experiments involving four mice/group; **P < 0.01; ***P < 0.001; Student *t *test. **(E) **Properdin-deficient arthritic neutrophils isolated from blood have higher C5aR expression than do wild-type cells, as shown on the histograms. The data on the graph are expressed as the mean ± SD of Ly6G^+^C5aR^+ ^cells from three experiments involving four mice/group. *P < 0.05; ***P < 0.001; Student *t *test. **(F) **C5L2 was expressed on human blood CD16^+ ^cells but not on mouse blood Ly6G^+ ^WT cells when compared with the isotype controls of mouse IgG2a, for human C5L2 antibody and of rat IgG2b, a control for mouse C5L2 antibody. The data are representative of three separate experiments involving four mice/group.

At day 10 of CAIA, we observed increased frequencies of Ly6G^low^CD11b^+ ^and Ly6G^high^CD11b^+ ^cells in BM (Figure 2A) and of Ly6G^+^CD11b^+ ^in blood of WT compared with KO CAIA mice (WT PBS group, 5.4% ± 0.9; KO PBS group, 4.2% ± 1.0; WT CAIA group, 35.8% ± 1.2; KO CAIA group, 14.0% ± 5.0; *n *= 4; *P *< 0.05). BM and blood Ly6G^+^CD11b^+ ^cells were stained with antibodies against RANKL (Figure 2B, D) and C5aR (Figure 2C, E). Surface RANKL was detected on BM WT but not on BM KO CAIA cells (Figure 2B). The molecule was barely expressed on mouse blood KO cells, and we found fewer Ly6G^+^RANKL^+ ^cells in KO CAIA mice (Figure 2D). BM cells from WT and KO mice expressed C5aR (Figure 2C) to a similar extent. Interestingly, we found higher C5aR expression and more C5aR-positive cells within the Ly6G^+ ^blood population of KO than in that of WT mice (Figure 2E).

Regarding C5L2, another receptor for C5a, we were not able to detect its expression on mouse blood Ly6G^+ ^in contrast to human peripheral CD16^+ ^cells (Figure 2F). As CAIA is a FcγR-dependent experimental model, we analyzed the expression of FcγR on synovial and blood neutrophils and on monocytes with flow cytometry (see Additional file 1). Additional file 1 shows higher FcγR expression and increased frequencies of Ly6G^+ ^FcγR^+ ^cells in WT CAIA mice in comparison to the KO CAIA group (Additional file 1A). Circulating CD14^+ ^KO CAIA monocytes expressed more FcγR than did those in WT CAIA mice (Additional file 1B). Synovial Ly6G^+^CD11b^+ ^cells with elevated FcγR expression were found in WT and KO CAIA mice when compared with PBS-injected control groups (Additional file 1C).

### Altered production of proinflammatory cytokines by splenic CD4^+ ^T cells in properdin-deficient mice with CAIA

**Figure 3:**
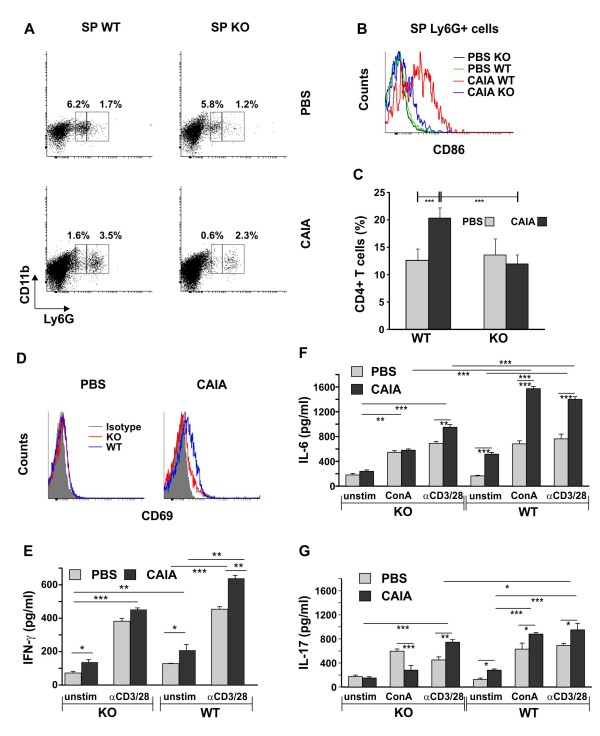
**Frequencies of neutrophils and CD4^+ ^T cells in spleen of properdin-deficient and wild-type mice**. **(A) **Ly6G^low^CD11b^+ ^and Ly6G^high^CD11b^+ ^cells were determined in spleen of properdin-deficient and wild-type mice. The representative experiment shows increased frequencies of splenic Ly6G^high^CD11b^+ ^cells in arthritic mice. **(B) **Elevated expression of co-stimulatory molecule CD86 on wild-type arthritic Ly6G^high^CD11b^+ ^cells. The histograms are representative of three separate experiments involving five mice/group. **(C) **Flow-cytometry analyses of splenocyte population demonstrated increased percentage of CD4^+ ^T cells in wild-type arthritic mice compared with the properdin-deficient CAIA group. The mean ± SD of the CD4^+ ^T cells in spleen are presented on the graph, ***P < 0.001; Student *t *test. **(D) **Activation marker CD69 was barely expressed on the arthritic wild-type and properdin-deficient CD4^+ ^T splenic populations. The histogram is representative of three separate experiments involving five mice/group. The ability of splenic CD4^+ ^T cells to secrete **(E) **IFN-γ, **(F) **IL-6, and **(G) **IL-17 was determined with ELISA. Cells (1 × 10^6 ^cells/ml) were isolated from PBS or CAIA groups at Day 10 of disease and were stimulated in 48-well plates with plate-bound anti-CD3 (10 μg/ml) and soluble anti-CD28 (5 μg/ml) antibodies (αCD3/28) or with ConA (2 μg/ml; where indicated), and in the presence of murine recombinant IL-2 (10 ng/ml). A control group of nonstimulated cells (unstim) was included in the experiments. After 48 hours of culture, supernatants were collected for cytokine determination. Data represent the mean ± SD. *P < 0.05; **P < 0.01; and ***P < 0.001; Student *t *test.

In our study, Ly6G^high^CD11b^+ ^cells were between 1% and 2%, whereas Ly6G^high^CD11b^+ ^cells were 5% to 6% in both KO and WT spleen populations (Figure 3A). At day 10 of CAIA, the frequencies of Ly6G^high^CD11b^+ ^cells increased two times, and fewer Ly6G^low^CD11b^+ ^cells were detected in the spleen (Figure 3A). Interestingly, Ly6G^high^CD11b^+ ^cells upregulated CD86 expression only in the WT CAIA group but not in KO CAIA mice (Figure 3B). CD86 is an important costimulatory molecule for T cells. Flow-cytometry analyses of the splenocyte population demonstrated an increased percentage of CD4^+ ^T cells in WT CAIA mice compared with the KO CAIA group at day 10 of disease (Figure 3C). Activation marker CD69 was barely expressed on the arthritic WT and KO CD4^+ ^T splenic population (Figure 3D). WT CAIA cells spontaneously release higher amounts of IFN-γ (Figure 3E), IL-6 (Figure 3F), and IL-17 (Figure 3G), whereas KO CAIA cells secrete IFN-γ but not IL-17 and IL-6 (Figure 3F, G). Both WT and KO cells responded to anti-CD3/anti-CD28 stimulation with markedly increased cytokine production. However, the amounts of secreted cytokines by WT CAIA cells were higher than those of the KO CAIA ones. Of note, ConA induced vigorous IL-17 and IL-6 production by WT CAIA but not by KO CAIA cells (Figure 3F, G).

### Altered phenotype of blood CD4^+ ^T cells in properdin-deficient mice with CAIA

**Figure 4:**
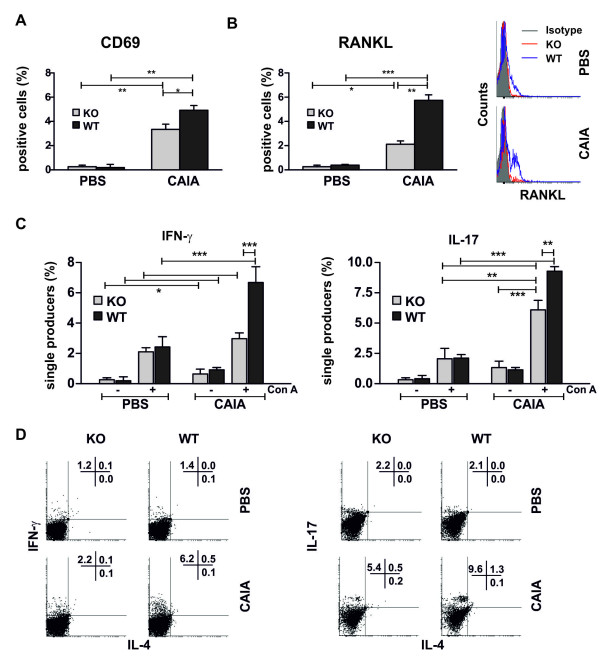
**Phenotype of blood CD4^+ ^T cells in properdin-deficient and wild-type mice**. **(A) **Peripheral CD4^+ ^T cells from arthritic properdin-deficient mice showed lower expression of CD69 compared with WT CAIA lymphocytes. Data represent the mean ± SD of positive cells from three experiments involving five mice/group. *P < 0.05; **P < 0.01; Student *t *test. **(B) **CD4^+ ^RANKL^+ ^T cells in blood of CAIA KO mice were fewer than those in the CAIA WT group. The data are expressed as the mean ± SD of positive cells from three experiments involving five mice/group.*P < 0.05; **P < 0.01; and ***P < 0.001; Student *t *test. Histograms show RANKL expression on CD4^+ ^T cells from properdin-deficient and wild-type mice in one individual experiment. **(C) **Blood CD4^+ ^T cells from wild-type mice were more sensitive to Con A stimulation than were those from properdin-deficient mice and had significantly higher intracellular levels of IFN-γ and IL-17. CD4^+ ^T cells were isolated at Day 10 of CAIA and stimulated with Con A (2 μg/ml). After 48 hours of culture, cells were restimulated with PMA and ionomycin and subjected to flow-cytometry analyses for the expression of IL-17 and IFN-γ. Data represent the mean ± SD of single IL-17 or IFN-γ producers of three experiments involving five mice/group. *P < 0.05; **P < 0.01; ***P < 0.001; Student *t *test. **(D) **One representative experiment showing the lack of significant IL-4 production in Con A-stimulated CAIA KO and WT cells. The intracellular level of IFN-γ and IL-17 in activated arthritic properdin-deficient CD4^+ ^T lymphocytes was lower than that in the CAIA WT group.

The initial inflammatory process involving complement and blood neutrophils and monocytes results in the activation of peripheral CD4^+ ^T cells. Blood CD4^+ ^T cells expressing the early activation marker CD69 were fewer in KO CAIA mice in comparison with the WT group (Figure 4A). The lack of properdin resulted in markedly inhibited RANKL expression on CD4^+ ^T cells and reduced numbers of CD4^+ ^T RANKL^+ ^cells in the periphery (Figure 4B).

In RA patients, CD4^+ ^T cells in the periphery are prone to produce proinflammatory cytokines. Thus, we determined the intracellular level of IFN-γ and IL-17 in blood CD4^+ ^T cells (Figure 4C). Nonstimulated CD4^+ ^T cells from control KO and WT mice showed a low production of IFN-γ and IL-17 that was upregulated by Con A stimulation (Figure 4C). However, no significant difference in the frequencies of single IFN-γ- and IL-17-producing cells between Con A-stimulated control KO and WT cells was observed. Nonstimulated control CD4^+ ^T lymphocytes showed weaker IFN-γ and IL-17 production than did nonstimulated arthritic cells (Figure 4C). CAIA WT CD4^+ ^T cells were more sensitive to Con A stimulation than were those from KO mice, because they had significantly higher intracellular levels of IFN-γ and IL-17 (as shown on Figure 4C and in one representative experiment in Figure 4D).

Concerning Th2 cytokine IL-4, its intracellular level was low in nonstimulated and ConA-stimulated CD4^+ ^T KO and WT mice (Figure 4D).

### Abrogated RANKL-dependent osteoclast differentiation of properdin-deficient BM precursors

**Figure 5:**
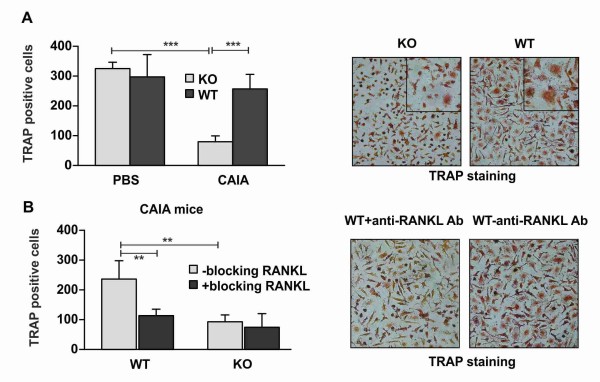
**Osteoclast differentiation of properdin-deficient and wild-type bone-marrow precursors**. **(A) **BM cells from control and CAIA mice (WT and KO) were differentiated for 7 days, and the number of mature osteoclasts was determined after specific TRAP staining. The graph shows the mean ± SD of TRAP-positive cells from three experiments with three sample repeats/group. ***P < 0.001; Student *t *test. One representative experiment is shown on the photos captured by a Nikon camera at a magnification of ×100 and for photo insert of ×400 of light-microscope lens. **(B) **TRAP-positive cells in BM cultures generated in the presence of blocking antibody against RANKL (blocking RANKL). The graph shows the mean ± SD of TRAP-positive cells in CAIA WT and KO cell cultures from three separate experiments with three sample repeats/group. **P < 0.01; Student *t *test. One individual experiment is shown on the photos taken at magnification ×100 with a light-microscope lens.

Our data showed that RANKL is expressed to a lesser extent by populations in BM (Ly6G^+ ^cells) and in the blood of KO mice (neutrophils and CD4^+ ^T cells), suggesting altered RANKL-dependent processes in conditions of properdin deficiency. Thus, we next evaluated the RANKL-mediated osteoclastogenesis of BM cells isolated from control and KO and WT CAIA mice. The specific TRAP staining showed similar numbers of generated osteoclasts in control KO and WT cultures (Figure 5A). Osteoclast differentiation of properdin-deficient BM CAIA cells was inhibited, and fewer TRAP-positive cells were detected in these cultures. We suggest that BM precursors from properdin-deficient CAIA KO mice were less sensitive to the action of RANKL.

We set up an experiment in which WT and KO CAIA BM cells were differentiated in the presence or the absence of a blocking antibody against RANKL (Figure 5B). We observed a reduced number of TRAP-positive cells in WT CAIA cell cultures, similar to that in the KO CAIA group. Osteoclast differentiation of properdin-deficient CAIA BM cells was not changed by blocking RANKL (Figure 5B).

### Immunohistochemical profiling of bone-related markers in arthritic joints

**Figure 6:**
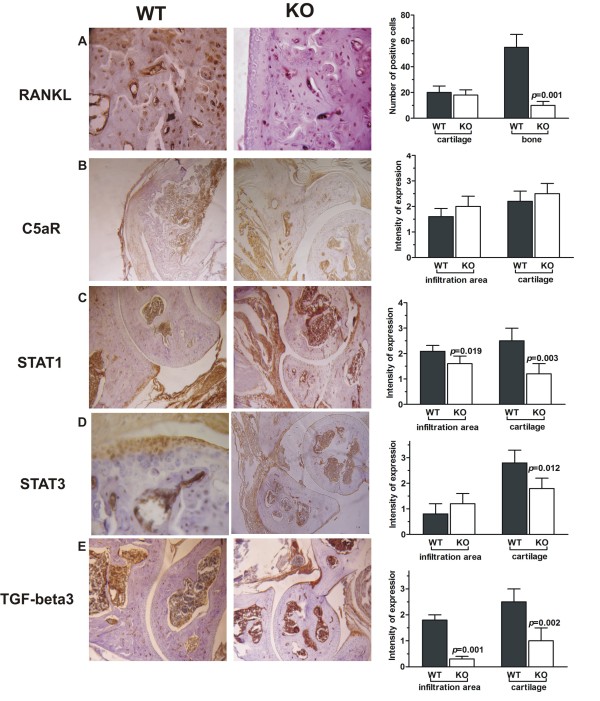
**Immunohistochemical profiling of bone-destructive markers in CAIA joints**. **(A) **Wild-type CAIA joints showed threefold higher expression of RANKL at Day 10 of disease than did those from KO CAIA mice. **(B) **CAIA WT and KO mice expressed C5aR in the infiltration area and in the bone. Properdin-deficient mice with CAIA had lower STAT1 **(C) **and STAT3 **(D) **expression in the infiltration area compared with the WT CAIA group. **(E) **TGF-β expression was decreased in the inflammatory area and the cartilage in properdin-deficient CAIA joints. The findings shown in (A) through (E) are representative of 10 sample sections from five mice/group; magnification ×40. The graphs show the mean ± SD of positive cell numbers (A, for RANKL expression) or intensity expression (B through E), for STAT1, 3 and TGF-β3 expression) calculated after assessment of 10 sections from five mice/group. *P *values are according to the statistical analyses with Student *t *test.

At day 10 of CAIA, the joint sections were analyzed for the expression of C5aR and several markers of bone destruction (Figure 6). The molecule associated with bone resorption RANKL was expressed approximately threefold higher in the cartilage of WT arthritic mice than of KO CAIA mice (Figure 6A). C5aR-positive cells were observed in the infiltration area and cartilage of CAIA groups, with no significant difference between WT and KO animals (Figure 6B). STAT1-positive staining was strong in the WT infiltration areas and cartilage and less obvious in KO joints (Figure 6C). STAT3 expression was similar in the infiltration zones of WT and KO mice, whereas in cartilage, its staining intensity was inhibited in the CAIA KO group (Figure 6D). CAIA development in KO mice was accompanied with suppressed TGF-β expression in the joints (Figure 6E).

## Discussion

In the present study, we evaluated the role of properdin, the regulator of AP, in the development of CAIA. Arthritis was induced by injecting properdin-deficient and WT mice with a monoclonal antibodies cocktail optimized for the use in C57BL/6 animals. At day 10 of CAIA, we observed marked cellular infiltration in the synovium and moderate cartilage damage in WT mice. Immune complexes in CAIA activate metalloproteinases that cleave collagen and, in turn, induce cartilage matrix loss. We were not able to detect a significant PG depletion in WT and KO mice, but the CAIA model was followed up for 10 days, which, however, cannot exclude more severe PG degradation at later stages of disease. Cartilage loss during the progression of arthritis is accompanied by a process of cartilage repair. TGF-β is a factor that stimulates collagen II and PG synthesis and inhibits cartilage-degrading enzymes [32]. In the long term, decreased expression of TGF-β in KO CAIA joints may affect osteophyte formation and joint deformation. The progression of arthritis in KO mice is related to the downregulation of STAT1 in the joints. The role of the transcription factor for the inhibition of inflammation has been shown in STAT-1^-/- ^mice with exacerbated ZIA [33] and in mice with established arthritis treated with nanoparticles encapsulating STAT1-targeted siRNAs [34].

CAIA progression in KO mice is retarded, with milder clinical symptoms and weaker synovial cell infiltration, than that in WT mice. Similarly, properdin-deficient transgene Cfp^-/- ^mice develop less-severe K/BxN arthritis [35]. In this study, BM chimeras between WT and Cfp^-/- ^mice were generated. The authors found lower Cfp mRNA levels in BM Cfp^-/-^/WT chimeras than in WT/WT ones and suggest that the major source of plasma properdin is BM-derived CD11b^+ ^cells.

In our study, Ly6G^high^CD11b^+ ^KO and WT neutrophils expressed C5aR, and thus showed the ability to respond to C5a after their mobilization from the BM. At day 10 of CAIA in the BM KO population, we observed significantly decreased frequencies of mature Ly6G^high^CD11b^+ ^cells and of maturing Ly6G^low^CD11b^+ ^neutrophils. These data indicate an abnormal generation of Ly6G^+ ^cells that, in turn, resulted in lower frequencies of Ly6G^+ ^circulating cells in blood and synovial fluid of properdin-deficient mice. Therefore, Ly6G^+^CD11b^+ ^neutrophils can be considered a cellular phenotype related to properdin deficiency. In support of this notion, the studies show that neutrophils (a) secrete properdin from the intracellular depot [36,37], (b) bind the secreted properdin [38], enhancing the assembly of AP C5-convertase on the cell membrane generating C5a fragments [38]. These activities of neutrophils are regulated by TNF-α, and probably by other cytokines and factors in the microenvironment.

C5a, as a chemoattractant, regulates the infiltration and accumulation of neutrophils in the synovial fluid and maintains inflammation [39]. In CAIA KO mice decreased amounts of C5a in synovial fluid resulted in reduced numbers of C5aR^+^-bearing neutrophils. In the joints, C5a can bind its receptor expressed on synovial fibroblasts and chondrocytes. In the CAIA model, the lack of properdin did not change cartilaginous C5aR expression. However, our previous observations in ZIA showed that KO mice were able to upregulate C5aR expression in the joints at a later stage of disease (day 30) [28]. C5a binds to two receptors on neutrophils: C5aR (CD88) and C5L2 receptors [40]. In contrast to human CD16^+ ^cells, Ly6G^+^CD11b^+ ^blood neutrophils showed nonsignificant surface expression of C5L2 when compared with the isotype control. However, arthritic blood Ly6G^+^CD11b^+ ^KO cells expressed more C5aR. Reduced serum C5a levels have been observed in naïve KO mice and in KO mice with acute inflammation and zymosan-induced arthritis [28,30]. Thus, increased C5aR expression is more likely due to insufficient engagement of C5a by blood neutrophils. We observed similar C5aR expression on WT and KO BM arthritic cells, which also indicates a modulation of receptor expression by C5a amounts in the periphery. The binding of C5aR to the ligand can modulate disease pathogenesis by regulating the balance of activating and inhibitory FcγR receptors [41,42]. In blood, the lack of properdin resulted in a significantly decreased FcγR expression on KO neutrophils and an increased surface FcγR on circulating CD14^+ ^cells (shown in Additional file 1). In our experiments the antibody clone 2.4G2 can recognize both FcγRIII and FcγRII receptors on KO neutrophils and monocytes. Thus, we could not exclude altered expression of the FcγRII isoform versus the FcγRIII on the same cells or a difference in the inhibitory FcγRIIb expression on the particular cell type, as shown by Bruhns *et al. *[43]. CAIA induction also involves antibodies of different IgG isotypes that can be recognized by specific FcγR receptors. Our data presume a complex interplay of activating and inhibitory FcγR receptors in condition of properdin deficiency and give a background for future investigations in this direction.

RANKL is responsible for osteoclast differentiation, activation, and survival, and drives bone resorption and bone erosion. In CAIA mice, we found retarded disease progression and decreased RANKL in cartilage. Moreover, BM precursor cells from CAIA KO mice differentiated poorly to mature osteoclasts in the presence of RANKL. In contrast to CAIA WT cultures, the blocking RANKL antibody did not affect the numbers of TRAP-positive CAIA KO cells, indicating a decreased sensitivity of KO BM precursors to RANKL signaling. RANKL interactions during osteoclast formation in IL-1β conditions can be regulated by C3a and C5a [44], because BM cells express C3aR and C5aR [45]. Recently, it was shown that C3^-/- ^BM cells exhibit lower RANKL/osteoprotegerin expression ratios, produce less M-CSF and IL-6, and generate fewer osteoclasts than wild-type BM cells [46]. Both this study and our results suggest that the process of osteoclast differentiation is sensitive to the abrogation of complement AP. *In vivo*, this process is complex and can involve BM precursors of osteoclasts, cells that produce, express, and secrete RANKL and cytokines. In CAIA WT mice, Ly6G^+^CD11b^+ ^BM cells expressed RANKL and, together with other BM precursors, can migrate and accumulate in the synovium in response to generated C3a, C5a, and IL-17. Moreover, IL-17 can mobilize stem cells in mice with short- and long-term reconstituting capacity [47]. Osteoclastogenesis can be initiated by these RANKL-positive cells, which enrich the microenvironment. In the present study, we found CAIA WT synovial neutrophils expressing RANKL. This is in line with the observations showing the RANKL expression on synovial neutrophils in RA patients [24]. Contradictory results were obtained by Yeo *et al. *[25] demonstrating that synovial RA neutrophils do not express significant RANKL mRNA levels compared with B and T cells. This discrepancy can be due to the generally restricted transcription in neutrophils compared with other cell types. In synovial fluid, activated neutrophils produce proteases that can cleave membrane RANKL on them or on infiltrating T cells, which in turn, can maintain a high level of soluble RANKL.

In our study, downregulated RANKL expression was observed on synovial, peripheral neutrophils, and on blood CD4^+ ^T cells from CAIA KO mice. The cytokine microenvironment is likely to reduce the surface RANKL on KO immune cells. Proinflammatory cytokine IL-17 is present in the synovium and serum of RA patients [48]. IL-17 regulates osteoclast differentiation and favors the activation of synovial fibroblasts and neutrophils. In our study, we found diminished IL-17 levels along with decreased numbers of RANKL-positive Ly6G^+^CD11b^+ ^cells in the synovial fluid of properdin-deficient mice. In both strains, WT and KO, synovial arthritic neutrophils were able to express FcγR when IL-17 was present in the fluid. It has been shown that IL-17 can enhance cartilage destruction in immune-complex-mediated arthritis by increasing the local numbers of FcγR-bearing neutrophils [49].

The lack of properdin slightly reduced the amounts of plasma IL-17 in CAIA mice. Peripheral CD4^+ ^T lymphocytes showed a decreased ability to produce IL-17 and IFN-γ in response to Con A stimulation *in vitro*. Concerning Th2 cytokine IL-4, its intracellular level was low in nonstimulated and ConA-stimulated CD4^+ ^T cells from KO and WT CAIA mice. However, IL-4 can have both a detrimental and a protective role in CAIA, because IL-4-deficient mice are protected from the disease [50].

Several reports have shown enhanced T-cell activation and differentiation as a result of a direct interaction between blood neutrophils and T cells [51,52]. In CAIA KO mice, we found fewer Ly6G^+^CD11b^+ ^cells in the blood and a decreased ability of CD4^+ ^T cells to produce IL-17. CAIA KO neutrophils also showed upregulated C5aR compared with WT cells. More recently, the essential role of C5aR for Th17 cells was described [53]. In this study, C5aR deficiency in SKG mice inhibited the expansion of Th17 cells. Th17 cell differentiation, however, requires TLR4, IL-6, and complement interactions [54]. The complement effect on Th17 cells is dependent on C5a-receptor expression and physiologically relevant levels of C5a [54]. In KO mice, C5a levels are reduced during arthritis progression [28] that can contribute (a) to limited engagement of C5aR on Ly6G^+ ^neutrophils, and/or (b) a failure in Th17 differentiation. When neutrophils are in more-dense contact with CD4^+ ^T cells, as in the spleen, they can provide co-stimulatory signals promoting T-cell differentiation. Co-stimulation can be enhanced by complement fragments. Impaired activation of naïve CD4^+ ^T cells by CD80^-/-^, CD86^-/-^, and CD40^-/- ^antigen-presenting cells is reconstituted by locally presented C5a or C3a [55]. CAIA developed with increased frequencies of Ly6G^high ^cells in the WT and KO spleen. In contrast to KO CAIA cells, arthritic Ly6G^high ^WT neutrophils expressed CD86 that can promote T-cell activation and proliferation and contribute to the increased numbers of splenic CD4^+ ^T and the spontaneous secretion of pro-inflammatory cytokines IL-17, IFN-γ, and IL-6. KO CAIA CD4^+ ^T cells may receive fewer co-stimulatory signals from Ly6G^high ^neutrophils, sufficient for IFN-γ production, but not for IL-17 and IL-6 secretion. This altered T-cell function in KO mice can be sustained during arthritis progression, inducing changes in the phosphorylation of transcription factors or further responsiveness to restimulation.

## Conclusions

In the present study, the deficiency of properdin caused functional changes in both neutrophils and CD4^+ ^T cells that prevent the development of inflammatory processes and joint alterations. The lack of this regulator of the alternative complement pathway resulted in (a) a decreased RANKL expression on immune cells, (b) a reduced ability of blood and splenic CD4^+ ^T cells to produce pro-inflammatory cytokines, and (c) abrogated RANKL-dependent differentiation of bone marrow precursors to mature osteoclasts. Taken together, these results point on a new role of properdin in immune complex-induced arthritis and give more ideas for the design of novel therapeutic approaches in rheumatic diseases.

## Abbreviations

Ab: antibody; AP: alternative complement pathway; BM: bone marrow cells; CAIA: collagen-antibody-induced arthritis; CIA: collagen-induced arthritis; Con A: concanavalin A; C5aR: C5a receptor; ELISA: enzyme-linked immunosorbent assay; EDTA: ethylenediamine-tetraacetic acid; H&E: hematoxylin and eosin; FcγR: Fc gamma receptor; IFN: interferon; Ig: immunoglobulin; IL: interleukin; KO mice: properdin-deficient mice; LPS: lipopolysaccharide; M-CSF: macrophage colony-stimulating factor; MBL: mannose-binding lectin; MFI: mean fluorescence intensity; PG: proteoglycan; PMA: phorbol-12-myristate-13-acetate; RA: rheumatoid arthritis; RANKL: receptor activator of nuclear factor kappa B ligand; RNA: ribonucleic acid; SF: synovial fluid; STAT: signal transducer and activator of transcription; TLR: Toll-like receptor; TGF: transforming growth factor; TNF: tumor necrosis factor; TRAP: tartrate-resistant alkaline phosphatase; WT mice: wild-type mice; ZIA: zymosan-induced arthritis.

## Competing interests

The authors declare that they have no competing interests.

## Authors' contributions

All authors contributed to the conception of the study. PD, LB, and VM performed *in vivo *and *in vitro *experiments; PD, NI, WS, and CS analyzed the results and made the figures; PD, NI, and CS designed the research and wrote the final draft of the article. All authors read and approved the final manuscript.

## Authors' information

LB and VM are PhD students at the Department of Immunology, Institute of Microbiology, Sofia, Bulgaria, and this work will be a part of their PhD theses.

## Supplementary Material

Additional file 1 **FcγR expression on synovial and blood neutrophils and on monocytes in properdin-deficient mice with CAIA**. **(A) **FcγR was expressed on blood wild-type Ly6G^+ ^cells but not on properdin-deficient cells, as shown in one individual experiment. Frequencies of Ly6G^+ ^FcγR^+ ^in blood at day 10 of disease are presented on the graph. Data are expressed as the mean ± SD of the positive cells from three experiments involving four mice/group; *P < 0.05; ***P < 0.001; Student t test. **(B) **Elevated numbers of CD14^+ ^FcγR^+ ^cells in blood of KO CAIA mice are shown on the graph. Data represent the mean ± SD of positive cells from three experiments involving five mice/group. *P < 0.05; **P < 0.01; and ***P < 0.001; Student *t *test. **(C) **FcγR expression was found on synovial neutrophils from arthritic properdin-deficient and wild-type mice. The histograms are representative of three separate experiments and show the analyses of the synovial cell pool from five mice/group. Click here for file
