# Supplementary figures and images for: Abrogated RANKL expression in properdin-deficient mice is associated with better outcome from collagen-antibody-induced arthritis
Source: Arthritis Res Ther. 2012 Jul 25;14(4):R173. doi: 10.1186/ar3926 (PMC3580567; doi:10.1186/ar3926)

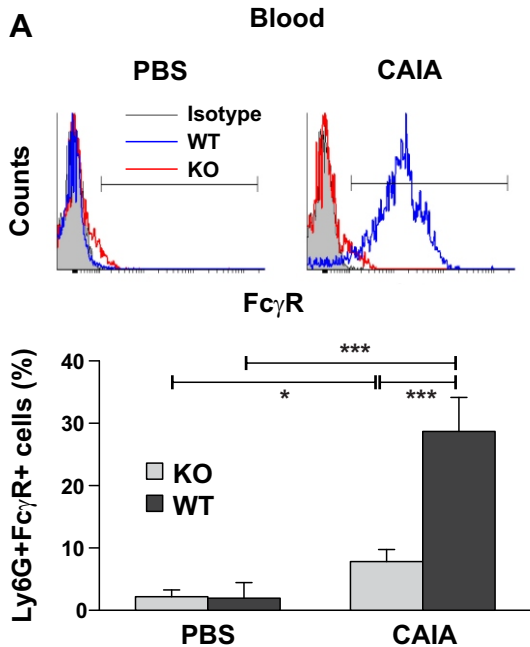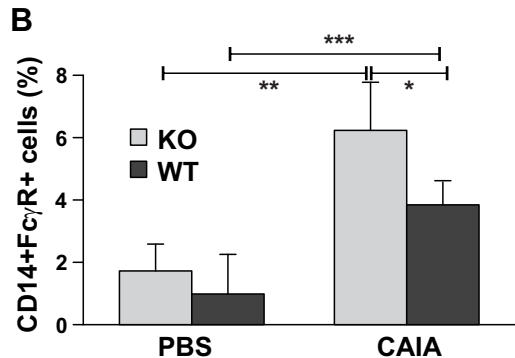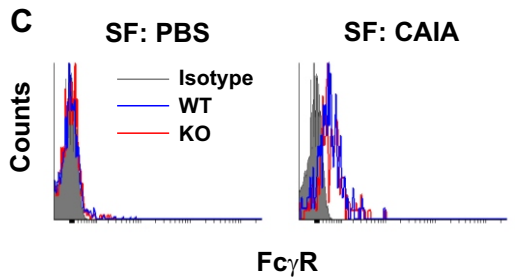

Supplement: Additional file 1 — FcγR expression on synovial and blood neutrophils and on monocytes in properdin-deficient mice with CAIA. (A) FcγR was expressed on blood wild-type Ly6G+ cells but not on properdin-deficient cells, as shown in one individual experiment. Frequencies of Ly6G+ FcγR+ in blood at day 10 of disease are presented on the graph. Data are expressed as the mean ± SD of the positive cells from three experiments involving four mice/group; *P < 0.05; ***P < 0.001; Student t test. (B) Elevated numbers of CD14+ FcγR+ cells in blood of KO CAIA mice are shown on the graph. Data represent the mean ± SD of positive cells from three experiments involving five mice/group. *P < 0.05; **P < 0.01; and ***P < 0.001; Student t test. (C) FcγR expression was found on synovial neutrophils from arthritic properdin-deficient and wild-type mice. The histograms are representative of three separate experiments and show the analyses of the synovial cell pool from five mice/group. [file ar3926-S1.PDF]
